# Differences in canine serum N-glycosylation pattern during infection and pregnancy

**DOI:** 10.1038/s41598-026-59457-z

**Published:** 2026-06-24

**Authors:** Margareta Ramström, Martin Lavén, Ragnvi Hagman, Ida Hallberg, Bodil S Holst

**Affiliations:** 1 Swedish Medical Products Agency, P.O. Box 26, Uppsala, 751 03 Sweden; 2 Department of Clinical Sciences, Swedish University of Agricultural Sciences, P. O. Box 7054, Uppsala, 750 07 Sweden

**Keywords:** Biochemistry, Biomarkers, Diseases, Immunology

## Abstract

Diagnosis of inflammatory disorders during canine pregnancy is complicated by changes caused by pregnancy. The total glycosylation pattern of body fluids reflects cellular status in health and disease. The aim of the present study was to evaluate the overall canine N-glycan pattern in dogs with infection compared to pregnant dogs. Serum samples from ten dogs with pyometra (bacterial disease), ten dogs with canine infectious respiratory disease, CIRD (primarily viral disease), ten pregnant dogs (day 24–30) and nine healthy non-pregnant dogs were included. A method optimised for N-glycan quantification in canine serum, based on RapiFluor-labelling and subsequent glycan mapping by HILIC-UPLC-FLR-MS was used. The levels of the two dominating glycans, A2G2S2 and FA2, and six glycan groups were evaluated. No significant differences between pregnant dogs and dogs with pyometra were observed. In dogs with CIRD and in healthy non-pregnant dogs, the levels of terminally galactosylated and high mannose glycans were higher, while the levels of glycan A2G2S2 were lower, than in pregnant dogs. The present study provides new insight into the canine N-glycan pattern during infection and pregnancy. Serum N-glycans and N-glycan groups do not seem suitable as biomarkers to differentiate inflammatory changes due to pregnancy from those caused by infections.

**Supplementary Information:**

The online version contains supplementary material available at 10.1038/s41598-026-59457-z.

## Introduction

Canine reproduction has specific features that differ from those in many other domestic animals. Dogs lack both a defined luteolytic factor and a known signal for maternal recognition of pregnancy. Relaxin is a hormone that increases in pregnant bitches^1^ but specific biomarkers that can be used during the first weeks of pregnancy to accurately differentiate pregnant and non-pregnant bitches are lacking. The identification of pregnancy-related infections during the first weeks of pregnancy is challenging since pregnancy causes biochemical alterations also seen during other inflammatory conditions^2^. During gestation, both circulating neutrophils and concentrations of acute phase proteins (APPs) rise^3–6^, a phenomenon also documented in bitches with pyometra^7,8^. Pyometra is a common uterine disorder associated with bacterial infection and progesterone-driven changes that occur during the luteal phase, the phase corresponding to pregnancy in non-pregnant dogs^9^. Because pyometra is potentially life-threatening, prompt diagnosis and treatment are essential. The most efficient and safest treatment is ovariohysterectomy. Given that this procedure results in irreversible infertility, accurately distinguishing between infection and normal pregnancy is vital, particularly in breeding animals^9^. Hence, diagnostic methods that can differentiate between normal gestation and other inflammatory states during early pregnancy, and preferably also clarify whether an infection is viral or bacterial, are needed.

Glycosylation, the enzymatic attachment of carbohydrate moieties, glycans, represents one of the most common and the structurally most diverse post-translational modification of serum proteins. In human medicine, analysis of the serum glycome has revealed disease-related shifts both in the abundance and structural variety of glycoproteins^10,11^. Key APPs such as alpha-1-acid glycoprotein (AGP), haptoglobin, and alpha-1-antitrypsin are glycosylated^10^. Changes in their circulating concentrations are established features of both canine pregnancy^3–6^ and pyometra^7,8^, as shown both in targeted analysis of individual proteins and by proteomic evaluations. Measurement of glycoprotein acetyls (GlycA) via nuclear magnetic resonance (NMR) has revealed an increase during pregnancy in dogs^12,13^. Such a measure can be regarded as describing a combined response of several glycoproteins.

Even though glycosylation and changes in glycosylation patterns have been extensively evaluated for human disorders, few studies addressing the overall N-glycan pattern in dogs have been presented to date^14–16^. The general N-glycome patterns in canine and human serum are similar, but dogs display non-human epitopes such as alpha-1,3-galactose (α-gal) and N-glycolylneuraminic acid (NGNA). In dogs infected with the parasite *Diroflaria immitis* (heartworm), an increase in galactosylation and core fucosylation, and a decrease in sialylation has been described^14^. In contrast, in a recent study of the N-glycosylation pattern during healthy canine pregnancy the opposite was described: sialylation increased and core fucosylation decreased^15^. The changes during canine pregnancy were similar to those reported for the human N-glycome during pregnancy^17,18^.

The aim of the present study was to evaluate whether the overall canine N-glycan pattern is affected in bitches with pyometra (a bacterial infection) and canine infectious respiratory disease (CIRD, kennel cough, most commonly a viral infection but may also be caused by bacteria or a combination of the two), and to evaluate if there are differences as compared to the N-glycan pattern in pregnant bitches. A method previously optimised for N-glycan quantification in canine serum^15^, based on RapiFluor-labelling^19^ and subsequent HILIC-UPLC-FLR-MS analysis, was used for analysis.

## Results

### N-glycans and N-glycan groups in canine serum

**Fig. 1:**
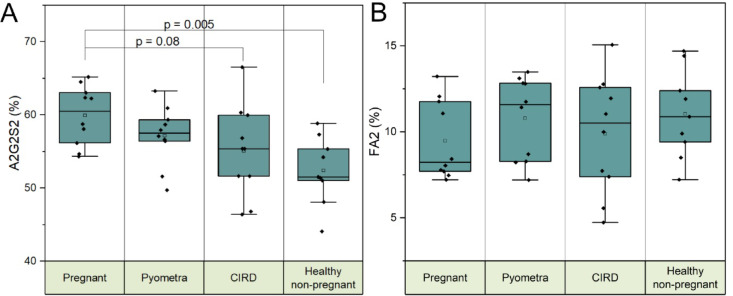
Abundance of glycan ASG2S2 (**A**) and glycan FA2 (**B**) in serum from pregnant (*n* = 10), pyometra (*n* = 10), CIRD (*n* = 10) and healthy non-pregnant (*n* = 9) dogs. The data is visualized by individual value plots and a boxplot where the box corresponds to data from the first to the third quartile (IQR), the bars represent 1.5 × IQR, the median is represented by a black line and the mean as a circle. Differences between pregnant dogs and the other groups were evaluated by one-way ANOVA followed by Dunnett’s correction for multiple comparison and all p-values < 0.1 are indicated.

**Fig. 2:**
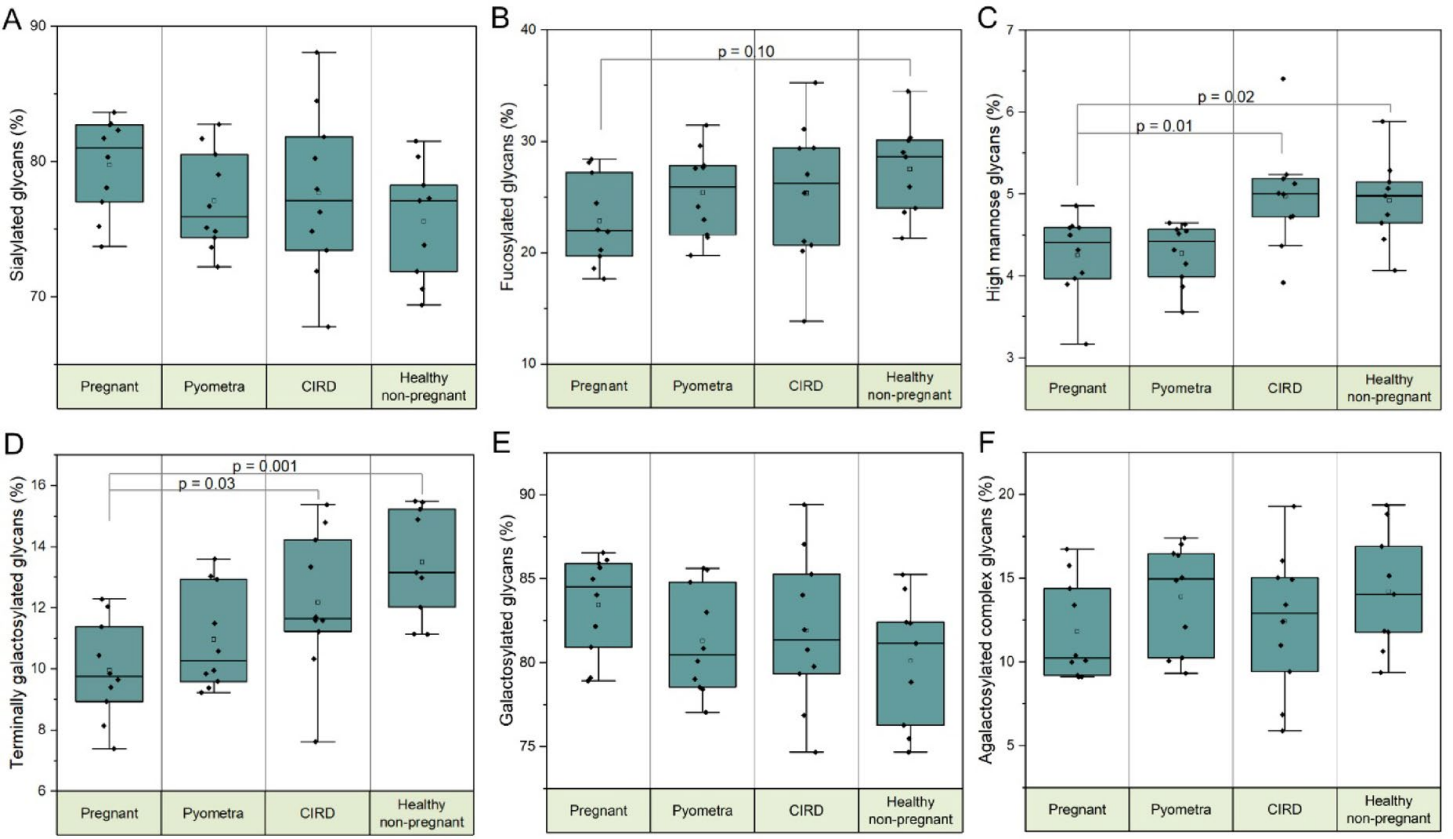
Abundance of (**A**) sialylated, (**B**) fucosylated, (**C**) high mannose, (**D**) galactosylated, (**E**) terminally galactosylated and (**F**) agalactosylated glycans in serum from pregnant, pyometra, CIRD and healthy non-pregnant dogs. The data is visualized by individual value plots and a boxplot where the box corresponds to data from the first to the third quartile (IQR), the bars represent 1.5 × IQR, the median is represented by a black line and the mean as a circle. Differences between pregnant dogs and the other groups were evaluated by one-way ANOVA followed by Dunnett’s correction for multiple comparison and all p-values < 0.1 are indicated.

In total, 39 dogs were included in the study: 10 pregnant dogs, 10 dogs with a bacterial infection (pyometra), 10 dogs with CIRD and 9 healthy non-pregnant dogs. The serum N-glycosylation pattern, including 50 assigned N-glycans, was evaluated for all 39 dogs. The abundances of the two dominating glycans, A2G2S2 and FA2, are presented in Fig. 1. In addition, the levels of six glycan groups, i.e., sialylated, fucosylated, high mannose, terminally galactosylated, galactosylated and agalactosylated complex glycans are shown in Fig. 2. The categorisation of each glycan is aligned with a previous study^15^ and described in Supplementary Table S1 The abundance of glycans A2G2S2 and FA2, and the six glycan groups are provided in Supplementary Table S2, for all individual dogs.

The levels of glycan A2G2S2 were lower in healthy non-pregnant dogs (*p* = 0.005) and dogs with CIRD (*p* = 0.080), as compared to pregnant dogs (Fig. 1). The corresponding levels were on average 7.5 and 4.8% points lower. The levels of A2G2S2 were found comparable for dogs with pyometra and pregnant dogs. No significant differences were observed for glycan FA2.

The levels of terminally galactosylated glycans were significantly higher for dogs with CIRD (*p* = 0.032) and healthy non-pregnant dogs (*p* = 0.001) as compared to pregnant dogs, differences of 2.2 and 3.5% points, respectively. Furthermore, high mannose levels were significantly higher, around 0.7% points, for CIRD dogs and healthy dogs (*p* = 0.023 and *p* = 0.011, respectively). The levels of fucosylated glycans were demonstrated to be higher in healthy non-pregnant than in pregnant dogs. No significant differences between pregnant dogs and dogs with pyometra were observed for the glycan groups included in the study.

### Biochemical analysis of samples

Since serum N-glycans are derived from glycoproteins, the overall serum protein repertoire could provide important information on differences and similarities in the glycan pattern. The results from routine biochemical analyses are summarised in Table 1. The study confirmed that the total protein concentrations were comparable for all four groups of dogs. The albumin concentrations were lower in dogs with pyometra as compared to healthy non-pregnant dogs. The concentrations of the acute phase protein CRP were significantly increased in pregnant dogs, dogs with pyometra and dogs with CIRD. The transferrin saturation was lower for pregnant dogs. Serum protein electrophoresis showed significantly higher levels of fractions alpha 2 and beta 2 in pregnant dogs and dogs with pyometra as compared to healthy non-pregnant dogs. Also, the proportion of fraction alpha 1 was higher in pregnant than in non-pregnant dogs.

**Table 1:** Total protein concentration and levels of biochemical serum analytes including albumin, C-reactive protein (CRP), total iron-binding capacity (TIBC), transferrin saturation and serum protein electrophoresis fractions for pregnant dogs, dogs with pyometra, dogs with CIRD and healthy non-pregnant dogs. For all analytes and categories, average values and range within brackets are provided. The corresponding reference range used in clinic is also shown. Statistical analysis was performed applying one-way ANOVA followed by Dunnett’s correction for multiple comparisons for normally distributed analytes and non-parametric Kruskal-Wallis test followed by Dunn’s test for non-normally distributed analytes. For all analytes, the other three groups were compared to healthy non-pregnant dogs; p-values < 0.1 are listed, NS denotes non-significant changes, # denotes non-normally distributed analytes.

Serum analyte		Clinical reference range	Category of dogs Average value [min-max range]			
			Pregnant (n = 10)	Pyometra (n = 9)	CIRD (n = 10)	Healthy non-pregnant (n = 8)
Total protein (g/L)		54–72	67.2 [62.6–72.9] NS	66.5 [58.0-80.9] NS	64.0 [54.9–75.1] NS	64.5 [58.0-69.5]
Albumin (g/L)		25–35	30.4 [26.5–33.2] NS	28.1 [21.6–33.7] p = 0.023	33.0 [29.5–36.5] NS	32.3 [24.5–35.0]
CRP (mg/L) #		< 7	68.6 [20–182] p < 0.0001	77.1 [3.6–271] p = 0.0008	22.6 [3.5–49] p = 0.026	4.3 [3.2–9.2]
TIBC (µmol/L) (49–95)		49–95	80.4 [73.3–96.4] NS	75.8 [47.2–98.5] NS	70.5 [55.6–88.0] NS	72.3 [65.3–81.2]
Transferrin saturation (%) #		23–72	22.4 [15.6–31.6] p = 0.025	32.8 [21.3–53.3] NS	32.6 [20.3–47.4] NS	31.9 [21.8–51.5]
Serum protein electrophoresis	Fraction alpha 1 (g/L)	0–2	2.26 [1.60–2.57] p = 0.045	2.00 [1.49–2.39] NS	1.78 [1.30–2.80] NS	1.89 [1.61–2.11]
	Fraction alpha 2 (g/L)	7–13	12.5 [9.00–15.0] p = 0.009	12.1 [7.49–15.1] p = 0.029	9.83 [6.82–16.4] NS	9.37 [6.44–11.5]
	Fraction beta 1 (g/L) #	2–5	3.92 [3.20–4.59] NS	4.18 [2.78–7.91] NS	3.40 [2.41–5.60] NS	3.56 [2.42–4.66]
	Fraction beta 2 (g/L)	6–12	9.78 [8.10–12.0] p = 0.079	11.0 [7.29–16.6] p = 0.004	7.84 [6.19–10.7] NS	7.78 [5.94–9.10]
	Fraction gamma (g/L) #	3–10	6.5 [4.60–9.28] NS	8.17 [3.98–21.6] NS	5.59 [3.70–7.32] NS	6.50 [4.21–8.85]

## Discussion

Pregnancy is an inflammatory condition, with systemically increased numbers of neutrophil granulocytes, and significantly elevated concentrations of several APPs. A recent study from our laboratory demonstrates that the overall serum N-glycan pattern changes during healthy canine pregnancy^15^. The aim of the current study was to investigate whether the overall N-glycan serum pattern of pregnant dogs differs from the N-glycan pattern in dogs with a bacterial infection (pyometra) and dogs with mainly viral infection (CIRD). For comparison, samples from healthy non-pregnant dogs were also included in the study.

In conclusion, no significant differences between pregnant dogs and dogs with pyometra were found. Thus, the overall glycosylation pattern in serum cannot be used to distinguish between these two conditions. In addition, the overall protein patterns as determined by routine biochemical analyses were similar for pregnant dogs and dogs with pyometra. In dogs with CIRD, the levels of terminally galactosylated and high mannose glycans were higher, while the level of glycan A2G2S2 was lower than in pregnant dogs. It is noted that the concentrations of all glycans and glycan groups varied greatly in the CIRD group, as indicated by the max-min ranges and individual value plots in Figs. 1 and 2. The CIRD group represents a heterogeneous group of dogs, and both viral and bacterial components may be responsible for the disease complex. The varying glycosylation patterns may be depending on type of microbial agent involvement. With respect both to biochemical attributes and the glycans and glycan groups, where differences were observed compared to pregnant dogs, similar glycan and protein concentrations were observed for CIRD and healthy non-pregnant dogs. CIRD is often a rather mild disease compared to pyometra. In the present study, 7 out of 10 dogs with CIRD had a mild disease. Milder inflammatory changes for dogs with CIRD is also reflected by the CRP concentrations that did not reach the same magnitudes as for pregnant dogs and dogs with pyometra.

Significant differences, both for individual N-glycans and N-glycan groups, were observed for pregnant dogs as compared to healthy non-pregnant dogs. The results were expected and in-line with the previous study^15^, demonstrating that the N-glycan changes associated with pregnancy are rather small, i.e. a few percentage points, but consistent. The differences between pregnant and healthy non-pregnant dogs determined in the current study were all of the same magnitude as previously reported, and the trends, i.e. increase or decrease were confirmed for all individual glycan groups. However, a limitation with the current study is the small sample size. Few dogs were included in the study and not all changes previously established were determined to be significant. Differences in sex and age of the dogs may also have influenced the result. The changes in the N-glycan pattern observed for pregnant dogs were quite the opposite to those reported for dogs infected by *Diroflaria immitis*^14^, which was characterised by an increase in the level of FA2 and a decrease in the glycan A2G2S2. It could be anticipated that the serum N-glycan patterns of dogs suffering from bacterial and viral infections would be altered in a similar way as for parasitic infections. However, the results from the current study demonstrate that the changes in N-serum glycan pattern for dogs with pyometra are more similar to the changes observed for pregnant dogs than to those with parasitic infection^14^, including an elevated level of glycan A2G2S2 as compared to healthy non-pregnant dogs. Serum FA2 originates to a large extent from IgG, which is one of the major components of fraction gamma in serum protein electrophoresis. Since IgG concentrations were around 1.5 times higher in dogs infected with *D. immitis*, the authors concluded that the IgG concentration is likely responsible for at least part of the change in the relative abundances of serum N-glycans. Our results indicate a slight increase in fraction gamma for dogs infected by pyometra, in line with a previous study^20^. Nevertheless, this change was not accompanied by an increase in FA2 abundance.

Changes in the overall glycan pattern could be due both to changes in the relative abundance of glycoproteins in serum and to modification of the glycan structures, i.e. the glycosylation microheterogeneity, of one or several glycoproteins. It is beyond the scope of this study to further investigate the main cause of the changes. However, several routine biochemical analyses were performed to investigate the protein repertoire of the dogs included in the study. The total protein levels were comparable for all four groups of dogs, on average around 65 g/L. Albumin is a negative APP, and the albumin levels were lower in dogs with pyometra than in healthy non-pregnant dogs. Decreased albumin levels is a common finding in dogs with pyometra^9^. However, albumin is not a glycoprotein, and differences in concentrations between dogs would not influence the N-glycan levels.

Transferrin is an abundant glycoprotein in serum of dogs and other mammals^10^. Transferrin saturation was lower in pregnant than in healthy non-pregnant dogs. Serum protein electrophoresis showed higher levels of the alpha1, alpha2 and beta2 fractions for both pregnant dogs and dogs with pyometra, confirming an acute phase response, as previously shown in the disease^20^. All three fractions are also known to contain abundant glycoproteins including alpha-1-antitrypsin, ceruloplasmin, alpha_2_-macroglobulin, haptoglobin and beta-2-glycoprotein^21^. Thus, glycoprotein levels were demonstrated to be altered both in pregnant dogs and dogs with pyometra, and the results from serum protein electrophoresis were quite similar for dogs of these two groups.

This is the first study to compare the total serum N-glycation pattern in pregnant dogs and dogs with infection. It was not possible to distinguish pregnant dogs from dogs with pyometra, and their acute phase responses were also similar. For dogs with CIRD, the acute phase response was not as pronounced, the N-glycosylation pattern varied more, and was for many attributes more similar to the pattern observed in healthy non-pregnant dogs. The previously described N-glycosylation pattern in dogs infected by *D. immitis* suggests that glycosylation poses an opportunity to differentiate inflammatory changes caused by infection from those caused by pregnancy. However, in clinical practice bacterial infections are more challenging to differentiate from pregnancy than parasitic infections, and according to the present study the N-glycosylation pattern is not well suited for this. In human medicine, glycosylation of specific proteins such as fibronectin^22^ and IgG^23^ differs with pathological conditions during pregnancy, and studies focusing on glycosylation of specific proteins are potentially valuable also for dogs.

## Methods

### Materials

GlycoWorks RapiFluor-MS N-Glycan Kit was purchased from Waters (Milford, MA, USA). Ammonium formate, eluent additive for LC-MS, LiChropur, ≥ 99.0%, was obteained from Supelco. Formic acid 99% for LC-MS, used for mobile phase preparations, formic acid, puriss. p.a., ACS reagent, reag. Ph. Eur., 98%, used for SPE clean-up and acetonitrile, HiPerSolv CHROMANORM for HPLC LC-MS grade - suitable for UPLC/UHPLC were purchased from VWR Chemicals.

### Sampling of dogs

Stored serum samples from dogs that had previously been included in other studies were used. Samples were from dogs with pyometra (10), dogs with CIRD (10), pregnant dogs (10) and healthy non-pregnant dogs (9). The dogs with pyometra, pregnant dogs and control dogs were all sampled at the University Animal Hospital, Swedish University of Agricultural Sciences in Uppsala, Sweden. The dogs with CIRD were sampled at two animal hospitals and one animal clinic in Sweden. Necessary permissions were obtained for all samples (see “Ethical considerations”). From all dogs, blood was collected from the cephalic vein. Serum was prepared by a standardized protocol and stored in aliquots for 1–10 years in -70 °C, until analysis. Differences in storage time is not seen as a limitation since the N-glycosylation pattern in dog serum has been demonstrated to be stable during long-term storage at -80 °C for at least 25 years^14^.

The pregnant dogs (mean age 4.1 years) were sampled the day of ultrasound diagnosis 4 weeks after ovulation (day 24–28). For dogs with pyometra (mean age 8.7 years), a venous sample was obtained at admission, prior to treatment. The diagnosis was based on history, physical examination, diagnostic imaging, and laboratory results and intraoperative examination of the uterus and abdominal organs and verified by postoperative macroscopic and histopathological examination. The dogs with CIRD were eight male and two female dogs, mean age 4.1 years. Their disease was characterised as mild in seven dogs and moderate in three dogs. Samples had been analyzed with PCR for canine adenovirus type 2, canine herpesvirus, equine influenzavirus, parainfluenzavirus, canine coronavirus and with bacteriological culture for presence mainly of *Bordetella bronchiseptica*. From two of the dogs with moderate disease, parainfluenzavirus had been detected. The heathy non-pregnant control group consisted of six female dogs and three male dogs of various breeds, mean age 3.9 years. (Supplementary Table S3)

### Sample preparation and HILIC-UPLC-FLR-MS analysis

N-glycans were released from serum glycoproteins and labelled using a GlycoWorks RapiFluor-MS *N*-Glycan Kit, as described by Ramström et al.^15^. Analysis was performed using an Acquity LC-system, equipped with an Acquity FLR detector and an Acquity TQD MS detector, together with an Acquity BEH Glycan Amide column, 2.1 × 150 mm, 1.7 μm particles (Waters, Milford, MA, USA), using settings as described previously^15^. Confirmation of the identity of glycans was performed by comparing obtained glycan mass values with theoretical mass values and by using identification data generated previously^15^. Additionally, analysis using an LC-MS QTOF system (Agilent 6545 QTOF, Agilent, Santa Clara, CA, USA) was carried out to further strengthen the identity of glycan species.

Quantitative data, used for N-glycan calculations and comparisons, were generated from FLR chromatograms, using Area% of glycan peaks, as extracted by the chromatography software, i.e. Abundance (%). A reporting threshold of 0.05% was applied. To control the performance of the sample preparation and of the HILIC-UPLC-FLR system, a QC sample, i.e. a serum sample from a healthy non-pregnant bitch, was included for each set of 7 samples.

### Biochemical analyses

Routine biochemical analyses were performed on the archived samples at the Clinical Pathology Laboratory of the University Animal Hospital, Department of Clinical Sciences, Swedish University of Agricultural Sciences. The serum concentration of CRP was measured with an automated immunoturbidimetric method (Gentian cCRP, Gentian AS, Moss, Norway) validated for dog serum. Concentrations of protein, albumin, iron and unbound iron binding capacity (UIBC) were analyzed on the chemistry instrument Beckman Coulter DxC 700 AU with standard reagents from Beckman Coulter Diagnostics (Brea, California, US). Total iron binding capacity (TIBC) was calculated (iron + UIBC). Transferrin saturation was calculated (iron/TIBC)*100. Gel electrophoresis was performed with Sebia Hydrasys 2 (Sebia, Solna, Sverige).

### Statistical analysis

Statistical analysis was performed in Minitab©. Normal distribution of the glycans was confirmed using the Anderson-Darling normality test and one way ANOVA was applied to compare the glycan/glycan group levels in serum from pyometra (*n* = 10), CIRD (*n* = 10), pregnant (*n* = 10) and healthy dogs (*n* = 9). Post-hoc analysis was performed applying the Dunnett’s correction for multiple comparison, setting pregnant dogs as the reference group. All p-values < 0.1 are reported by numbers to allow for objective interpretation. While *p* < 0.05 is generally considered as statistically significant, *p* < 0.1 is considered an acceptable threshold in this exploratory study of small sample size.

The Anderson-Darling test for normality was performed also for all biochemical analytes. Total protein, albumin, TIBC and serum electrophoresis fractions alpha-1, alpha-2 and beta-2 were found to be normally distributed. For these analytes, one way ANOVA followed by post-hoc analysis by the Dunnett’s correction for multiple comparison was applied. CRP, transferrin saturation and serum electrophoresis fractions beta-1 and gamma were not normally distributed, and for these analytes the non-parametric Kruskal-Wallis test was performed followed by Dunn’s test for multiple comparison. For all comparisons, the category “healthy non-pregnant dogs” was defined as the reference group. Due to lack of sample, one dog with pyometra and one healthy non-pregnant dog were excluded from the analyses.

## Supplementary Information

Below is the link to the electronic supplementary material.

Supplementary Material 1

Supplementary Material 2

Supplementary Material 3

## Data Availability

Data generated or analysed during this study are for most parts included in this published article (and its Supplementary Information files). All datasets generated during and/or analysed during the current study are available from the corresponding author on reasonable request.
